# Comparative Viability and Functionality of Bio‐Jetted and Threaded Human Umbilical Vein Endothelial Cells

**DOI:** 10.1002/smsc.202500199

**Published:** 2025-07-25

**Authors:** Prasad Sawadkar, Ayad Eddaoudi, Dale Moulding, Suwan N. Jayasinghe

**Affiliations:** ^1^ The Griffin Institute ‐ Northwick Park Institute for Medical Research Northwick Park and St Mark's Hospitals, Y Block, Watford Road, Harrow Middlesex HA1 3UJ UK; ^2^ Division of Surgery and Interventional Science University College London 43‐45 Foley St London W1W 7TY UK; ^3^ Flow Cytometry Core Facility University College London Great Ormond Street Institute of Child Health/Zayed Centre for Research into Rare Disease in Children 20 Guilford Street London England WC1N 1DZ UK; ^4^ Light Microscopy Facility University College London Great Ormond Street Institute of Child Health 30 Guilford Street London WC1N 1EH UK; ^5^ UCL Department of Mechanical Engineering BioPhysics Group Centre for Stem Cells and Regenerative Medicine Institute of Healthcare Engineering Torrington Place London WC1E 7JE UK

**Keywords:** aerodynamically assisted bio‐jets/threads, angiogenesis assay, bio‐electrosprays, cell electrospinning, chorioallantoic membrane assay, flow cytometry, human umbilical vein endothelial cells

## Abstract

Advanced functional biological assays are critical for interrogating a process and/or material(s). The chicken embryo chorioallantoic membrane (CAM—an alternative in vivo system) and the angiogenesis assays, are two such well‐established biological assays. In these investigative studies, both these assays are employed, together with immunostaining for CD31, and a clinical readout, namely flow cytometry, to fully assess post‐treated human umbilical vein endothelial cells exposed to both electric field and nonelectric field driven spraying and spinning approaches in comparison to controls. Both these categories of directly jetting or threading living cells have significant implications, from their ability to directly reconstruct living 3D complex architectures. Such architectures have a plethora of applications spanning tissue reconstruction, to their exploration as organoids, spheroids to other complex models, for drug discovery and development, screening, to many other fields, extending to evolving food industry. The studies carried out herein demonstrate post‐treated cells are viable and are comparable to controls, as assessed via flow cytometry. Post‐treated cells exhibited comparable angiogenic sprouting and formed microtubules as seen via both immunohistochemistry staining with CD31, and the angiogenic assay, similar to controls. Finally, post‐processed cells, demonstrated their ability to form a microvasculature indistinguishable to controls, by way of the CAM assay. These results push forward these platform biotechniques, for their exploration in both the biomedical laboratory and clinic.

## Graphical abstract

Electrosprayed porous collagen architectures, were populated with human umbilical vein endothelial cells by way of either electric or nonelectric field driven jetting or threading microtechnologies. Jetted and threaded cells are interrogated via both flow cytometry and the angiogenesis assay. These cell‐bearing architectures were later stained for CD31 and assessed for promoting vascularization, to finally forming a vasculature through the CAM assay.

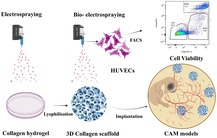

## 1 Introduction

Additive microtechnologies are increasingly playing a significant role in many areas of research and development, spanning the physical to the life sciences.^[^ ^1^ ^]^ In the context of their adoption to the biological and medical sciences, these additive microtechnologies, yielding the greatest interest/acceptance, would be those that have the ability to process large volumes of cells, in either single or multi‐cellular configurations (with the addition of other materials such as biomolecules etc.), without compromising the processed cells from a genetic level upwards, to finally possessing the ability to achieve its end application. For example, in the reconstruction of a living tissue, to do so with the least number of processing steps, thereby reducing human intervention, and the elimination of issues such as sterility, etc. To this end, additive biomanufacturing has evolved from those microtechnologies already in existence and widely explored in the physical sciences. These technologies range from the many manifestations of 3D printing,^[^ ^2^ ^]^ lab on chip (aka microfluidics),^[^ ^3^ ^]^ electric field (electrosprays and electrospinning),^[^ ^4^ ^]^ and nonelectric field (aerodynamically assisted jetting and threading)^[^ ^5^ ^]^ driven approaches. 3D printing, referred to as 3D bioprinting when handling biomolecules or cells directly has many manifestations, which vary based on their driving mechanism. They vary from, being driven by piezoelectricity, a solenoid, thermally to special extrusion (which could either be piston/air or screw driven) systems.^[^ ^6^ ^]^ Although in the physical sciences, many complex architectures have been successfully generated by these 3D printing systems, the technology is limited when handling advanced sensitive materials, such as biomolecules and living cells.^[^ ^7^ ^]^ In fact, the direct handling of biomolecules or living cells has resulted in either the biomolecules being damaged or the living cells undergoing cell death.^[^ ^7^ ^]^ This is directly a result of this technology, as it hinges on the inner bore diameters of the needles explored. Briefly, the inner bore diameter of the needles used in the many manifestations of 3D printing/bioprinting determines the generated residue sizes on deposition. Therefore, if residue sizes are to be limited, the processable material and/or cell concentrations (only carried out with low‐density‐single‐cell suspensions) have to be significantly reduced, to both reduce shearing within needles, and to the use of prefabricated substrates, which can reduce liquid spreading on deposition. Unfortunately, this operational obstacle cannot be overcome and therefore, the technology has undergone tempering in its capability to contribute to the biological and medical fields. From a biological standpoint, 3D bioprinted cells, have not undergone any interrogation using clinical readouts, such as flow cytometry, gene expression or cytogenetic studies. This unfortunately, limits confidence in the technology and its utility in both the biomedical laboratory and clinic.

Microfluidic‐based chips have been widely used in the handling of a comprehensive range of liquids in the chemical and physical sciences. These studies have elucidated the uniqueness of this class of microtechnology. In fact, this fluidic system is possibly the only microtechnology capable of generating a continuous and monodispersed distribution of droplets encapsulating a controlled quantity of either molecules and/or cells.^[^ ^8^ ^]^ Although the technology is a symphony to observe, the process has limitations in the quantities of processable materials (including cell suspensions), and the quantity of generated structures which are of practical use. That being said, the technology has been adopted as the flow system in cell and molecular analysis methods, for sequencing, etc. Note that in such systems, the required yield is nowhere near that demanded by real‐world applications, as in tissue engineering and regenerative medicine. Microfluidics has been explored in regenerative biology and medicine investigations; however, these are rather limited to the laboratory at present.^[^ ^9^ ^]^

Both 3D printing/bioprinting and microfluidics have carved out interesting and intriguing areas of research and development, but their utility with cells in the clinic is unfortunately, at present limited. Electrosprays and its sister technology, electrospinning, unlike 3D printing/bioprinting and microfluids, have been demonstrated in the physical sciences to handle large volumes of materials as viscous suspensions through either single/multiple needle systems with inner bore diameters of a few 1000 μm.^[^ ^10^ ^]^ Notably, yet possessing the capacity to generate droplets and residues from concentrated suspensions, in the few tens of nanometers to micrometer scale.^[^ ^10^ ^]^ Both electrosprays and electrospinning work on the principal of accommodating the flow of a liquid/suspension within a charged needle, held above a grounded electrode. The charged liquid on exiting the needle is accelerated toward the grounded electrode, via the electric field, during steady state conditions, a stream of droplets breaking off a jet or the jet elongating into a continuous fiber (collection over time generates a fibrous scaffold) is observed. The former is electrospraying and the latter is electrospinning. In both scenarios, the liquid properties play a critical role, in particular, liquid viscosity, along with many other media properties, together with both the equipment setup and operational settings. In both these techniques, the suspended material concentration and its dimensions play a critical role in the generated residues. Interestingly, these technologies are flexible in their ability to embrace multi‐coaxial needle systems for needle arrays having over 200 000 needles.^[^ ^11^ ^]^ Thus, scaling up is no issue. In the context of directly handling cells, both electrosprays and electrospinning have been demonstrated to handle a wide range of living cells (over 600 cell types) in either single and/or multiple cell type configurations, in concentrations of ≈10^7^ cells mL^−1^. These manifestations are now referred to as bio‐electrosprays (BES).^[^ ^12^ ^]^ and cell electrospinning (CE).^[^ ^13^ ^]^ Both these techniques have undergone extensive interrogation using clinical readouts such as cytometry, gene expression, and cytogenetic studies, demonstrating their ability to handle cells without damaging them from a molecular level upwards and indistinguishable to controls.^[^ ^14^ ^]^ The only comparable technology to both BES and CE is their nonelectric field driven counterparts referred to as aerodynamically assisted jets (AAJ) and threads.^[^ ^15^ ^]^ This is a technology born out of the developmental studies carried out on hydrodynamic focusing, explored in flow cytometry.^[^ ^16^ ^]^ Briefly, a needle holding the flow of a liquid is placed in a pressurized chamber which has an exit orifice in‐line and below the needle exit. The pressure within the chamber is much greater than the atmospheric pressure, thus creating a high‐pressure zone. Hence, the pressure gradient over the exit orifice creates a suction side which sees the drawing of the liquid from the needle through the exit orifice, subsequently undergoing break up into droplets or elongating into a continuous thread. The former is referred to in the physical sciences as AAJ, while the latter is known as aerodynamically assisted threads (AAT).^[^ ^15^ ^]^ Both AAJ and AAT have been interrogated similarly to both BES and CE to directly handle a wide range of living cells without damaging them from a molecular level upwards, and are indistinguishable from controls. These technologies are widely referred to as aerodynamically assisted bio‐jets (AABJ) and aerodynamically assisted bio‐threads (AABT).^[^ ^17^ ^]^ Note similar to both electrosprays/BES and electrospinning/CE, AAJ/AABJ and AAT/AABT possess the ability to generate fine residues comparable to those produced via both electrosprays/BES and electrospinning/CE, while processing large volumes/densities of suspensions. The only difference setting these two technologies apart is that one is electric field driven (electrosprays/electrospinning, BES/CE) and the other is not. The electric field has a significant effect on jet stability in the case of electrosprays/electrospinning, when processing highly conducting liquids, such as those containing metal particulates/biomolecules/cells. This processing obstacle is overcome by either using a coaxial needle system or a biofriendly polymer/low‐conducting viscous polymer. This issue is nonexistent for AAJ/AABJ and AAT/AABT as they are pressure driven and user safe.

Therefore, in these studies we chose to use human umbilical vein endothelial cells (HUVECs), as their functionality for forming blood vessels in a microvesculture, could be tested, together with their viability, post‐treatment with these microtechnologies. HUVEC's viability and functionality, was assessed with well established, advanced functional assays, immunostaining together with flow cytometry to fully study whether these microtechnologies, have any effects on the post‐treated cell viability, their ability to form microtubule networks, to finally assessing their ability to form a microvasculature, in an alternative in vivo system through the CAM assay in comparison to controls.

## 2 Results and Discussion

### 2.1 Scaffold Preparation

**Figure 1:**
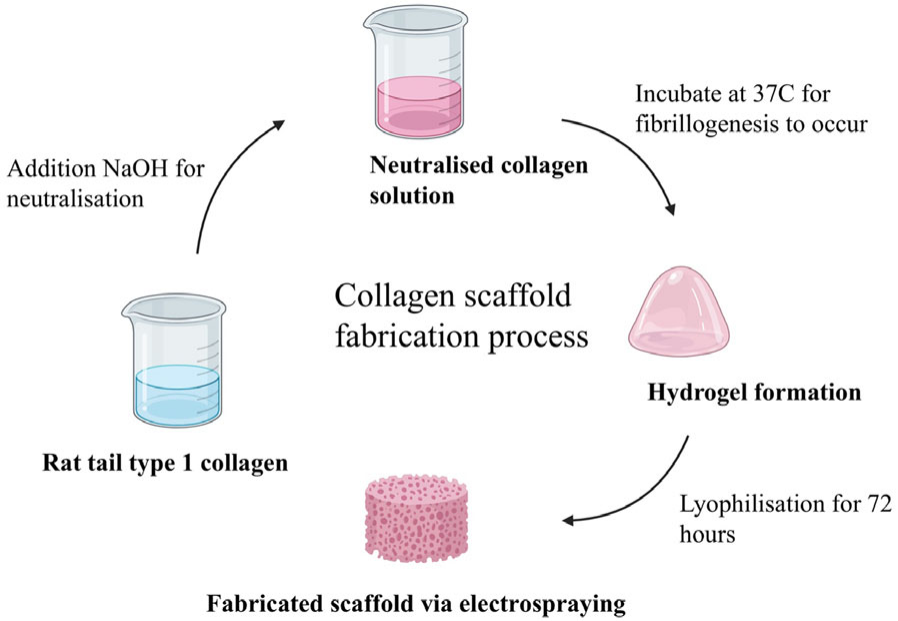
Steps for collagen scaffold fabrication. Figure created using BioRender.

**Figure 2:**
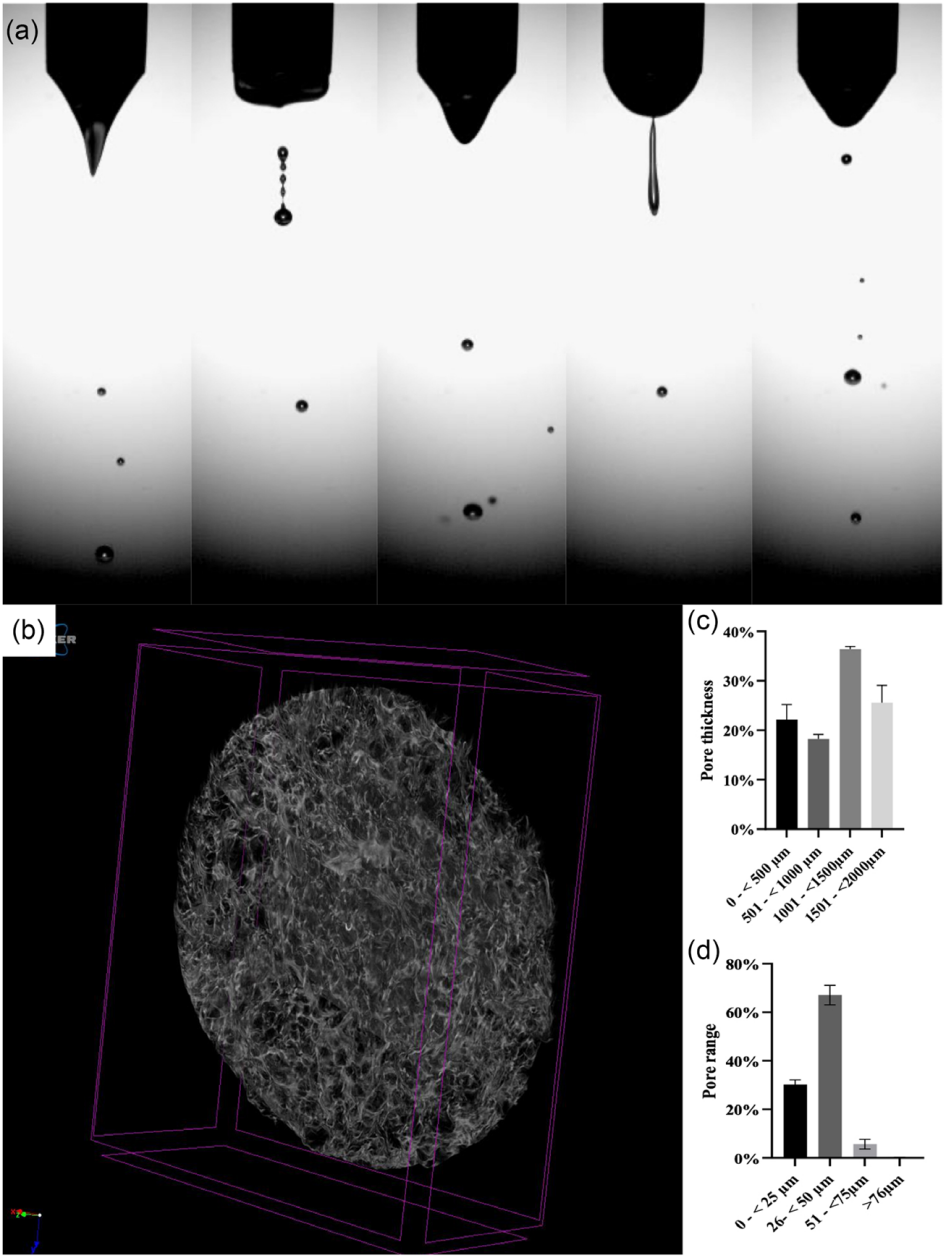
a) Representative digital high‐speed image sequence of the electrospraying of the collagen solution for scaffolding captured at 15 000 fps, b) characteristic micro‐CT of the 6 mm biopsy punched scaffold generated via electrosprays. Panels c,d) demonstrate scaffold characteristics in particular pore thickness and pore range, respectively.

Prior to bio‐jetting or threading HUVECs. We decided to generate collagen‐based scaffolds, which would act as substrates, onto which we would directly bio‐jet or bio‐thread the HUVECs. Note that these collagen‐based substrates would be biocompatible but will not be affected by the seeded cells remodeling. Our previous experience of processing cells with hydrogels was found to generate viable cell‐bearing hydrogel architectures, but would shrink over time, as a direct result of cell remodeling. This would be undesirable in these investigations, as we wish to assess the generated vasculature postseeding on CAM assays, to finally harvest them for analysis. Therefore, both jetting/threading systems were autoclaved for sterility and setup in a laminar flow safety hood as previously described.^[^ ^12^, ^13^, ^17^ ^]^ The collagen solution was prepared as described in the experimental section (**Figure** **1**). The prepared collagen solution was subjected to electrospraying and was found to spray in the unstable mode of jetting (**Figure** **2**a). Several electrosprayed 6 mm scaffold samples were generated and placed in a 96‐well plate. Subsequently, cell suspensions containing a HUVEC cell concentration of ≈1.2 × 10^6^ cells mL^−1^ were prepared for bio‐jetting and threading.

### 2.2 Scaffold Characterization

The micro‐CT analysis of the scaffolds demonstrated (Figure 2b–d) a varied distribution of pore thickness across different ranges, indicating the scaffold's heterogeneous structure. The pore thickness within the range of 0–500 μm was found to be 22.15 ± 3.06%. The pore thickness for the range of 501–<1000 μm was 18.21 ± 0.94%. Within the range of 1001–1500 μm, the thickness of the pores increased to 36.37 ± 0.56%. The pore thickness for the region of 1501–2000 μm was 25.55 ± 3.50%. In addition, the thickness of the pores was evaluated within narrower ranges. The pore thickness for pores ranging from 0 to 25 μm was found to be 30.22 ± 1.9%. Within the region of 26–<50 μm, the thickness of the pores exhibited a significantly higher value of 67.09 ± 3.97%. The pore thickness for the region of 51–75 μm was measured to be 5.66 ± 1.99%. Ultimately, when it comes to pores larger than 76 μm, the thickness of the pores was extremely small, measuring at 0.2 ± 0.1%. The results reveal significant disparity in pore thickness across several ranges, showcasing the scaffold's potential adaptability for a diverse array of clinical applications. The presence of relatively thick pores within the smallest size range (0–<25 μm) indicates a densely packed and potentially more condensed section of the scaffold. This characteristic could be advantageous for applications that necessitate a large surface area and intimate cell interaction, for example, in tissue reconstruction, cell therapy and the culturing of either single or multiple cell types in 3D.

The scaffold's major structural feature is reflected in the maximum pore thickness measured within the range of 26–<50 μm. This feature is anticipated to form optimal conditions for cell infiltration and nutrient flow, which are critical for tissue engineering applications. A notable pore thickness was observed in the intermediate ranges (501–<1000 and 1001–<1500 μm), indicating the scaffold's physical potential to accommodate various cell types in its niche and promoting cell growth and their extracellular matrix secretion. However, scaffolds showed a decrease in the pore thickness in the range of 1501–<2000 μm as compared to the intermediate ranges. This finding implies that the scaffold's design attempts to strike a balance between porosity and structural integrity. The highest pore range was >76 μm, which is rare, as indicated by the lowest thickness found. This distinctive characteristic may contribute to the overall mechanical properties of the scaffold. This scaffold's variety in pore characteristics indicates that it is perfect for complex tissue regeneration, repair and angiogenesis assays. The precise physiological responses to these pore size changes should be further studied in order to improve scaffold design for particular tissue engineering objectives.

### 2.3 Jetting and Threading HUVECs

**Figure 3:**
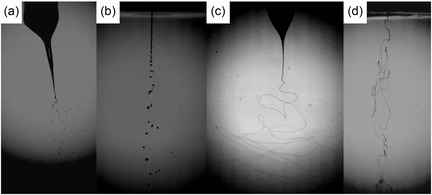
High‐speed digital images captured at 15 000 fps for a) BES, b) AABJ, c) CE, and d) AABT of HUVEC suspensions.

BES of the HUVEC suspensions was carried out at an applied voltage of ≈5 kV for a flow rate regime of ≈10^−9^ m^3^ s^−1^, in an electric field of 0.5 kV mm^−1^. Several samples were collected into sterile stainless steel petri dishes, during this process, for microscopy, flow cytometry for assessing cell viability, and those that were deposited directly onto scaffolds, in a 96‐well plate, respectively. Similarly, AABJ was carried out at an applied pressure of ≈1 bar for a flow rate of ≈10^−9^ m^3^ s^−1^, and samples were collected for microscopy, flow cytometry, and on scaffolds. In the case of both CE and AABT, we prepared a sterile polyvinyl alcohol (PVA) suspension at a concentration of 10 wt% and mixed it with the HUVEC cell suspension. This was required as the threading process in both these approaches requires the carrier medium to have a higher viscosity than complete cell media. Processing the PVA‐HUVEC suspension was found to form fibers during CE at an applied voltage of ≈4 kV for a flow rate regime of ≈10^−11^ m^3^ s^−1^, in an electric field strength of 0.4 kV mm^−1^. Collection for microscopy and flow cytometry was directly into sterile stainless steel petri dishes, while onto scaffolds was directly into a 96‐well plate containing the collagen scaffolds. Later, the PVA‐HUVEC suspension was exposed to AABT and collected for analysis similar to those CE samples. All bio‐jetted (BES/AABJ) and bio‐threaded (CE/AABT) HUVEC cell suspensions were at a concentration of ≈1.2 × 10^6^ cells mL^−1^. **Figure** **3** illustrates the bio‐jetting and threading process in action captured via a high‐speed digital camera system operating at 15 000 fps.

From Figure 3a, it is clear that the BES of HUVEC suspensions takes place in the unstable jetting mode, which is a direct result of the concentration of ions present and demanded by the cells metabolism, in suspension. Jet stability can be achieved if required, as previously demonstrated with the use of either a coaxial needle system or by suspending the cells in a biocompatible polymer.^[^ ^18^ ^]^ However, this is not the case for AABJ (Figure 3b) as the process is driven by a pressure gradient and thus jet stability is achieved in the single needle configuration without any complications whatsoever. Panels c) and d) in Figure 3 demonstrate the PVA‐HUVEC suspensions undergoing cell electrospinning and AABT respectively.

**Figure 4:**
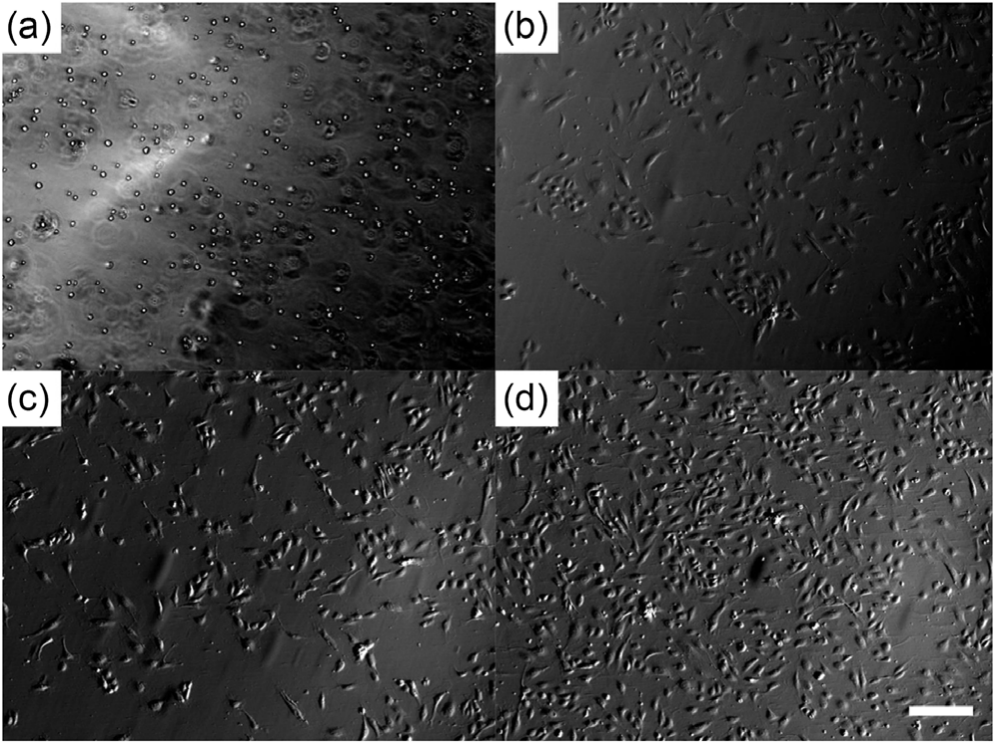
Representative micrographs depicting cells post‐bio‐electrosprayed at a) as collected, b) 24 hr, c) 48 hr, and d) 72 hr postcollection. Microscopy analysis of those CC (both HUVECs and PVA‐HUVECs not exposed to any treatment), and those samples exposed to CE, AABJ, and AABT were indistinguishable with those post‐bio‐electrosprayed samples. Scale bar in panel (d) is applicable to all panels and represents 330 μm.

Many samples were collected postprocessing for microscopy, for visually assessing cell viability and growth over a time course of 72 hr. **Figure** **4** shows micrographs of those BES samples cultured over the time course, which were indistinguishable from those samples collected for CE, AABJ/T and with controls (those untreated HUVEC cell suspensions).

Throughout these studies, we retained samples which were controls, namely those cell suspensions which were not exposed to any form of processing other than standard cell culture.

### 2.4 Assessing Cell Viability via Flow Cytometry

**Figure 5:**
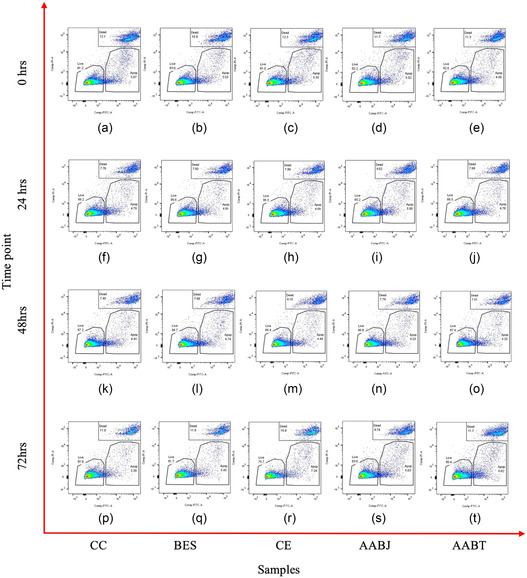
Representative dot plots of those cells interrogated via flow cytometry at the time course from day 0 (as jetted), 24, 48 to finally 72 hr. Panels a, f, k, p) are CC while, panels b, g, l, q) are bio‐electrosprayed, panels c, h, m, r) are cell electrospun, panels d, i, n, s) are aerodynamically bio‐jetted and finally panels e, j, o, t) are aerodynamically bio‐threaded samples.

During the bio‐jetting and threading process, several samples were collected from each processing technology for flow cytometry (**Figure** **5**). Each collected sample was equally split subsequently into four equal samples, which would allow the analysis of cell viability of the processed cells via flow cytometry, in comparison to controls over the 72 hr time course. The as bio‐jetted and threaded samples were aliquoted and labeled using the cell viability kit (FITC Annexin V Apoptosis Detection Kit with PI, BioLegend, 33 Greenwood Place London, NW5 1LB, UK) as per the manufacturer protocol and analyzed via an BD LSR II (Wokingham, Berkshire RG41 5TS, UK) flow cytometer. Figure 5 depicts dot plots identifying the cellular dynamics for those populations which are alive, those in early and late apoptosis and finally dead cells. Panels a–e represent those cellular dynamics of HUVECs a) culture control (CC), b) exposed to BES, c) cell electrospun, d) aerodynamically assisted bio‐jetted and finally, and e) AABT at day 0. Panels f–j represent those samples at the time point of 24 hr. Panels k–o are representative of those samples at the 48 hr time point, while panels p–t are representative of those cells at the 72 hr time point.

The flow cytometry dot plots shown in Figure 5, establish that jetted and threaded cells have no negative effects on the identified processed cell populations (live, apoptotic and dead cells) in comparison to controls. This is not unexpected, as we have seen and shown this via the analysis of a wide range of other cells through our previous investigations. Subsequently collected samples for each processing technology were assessed for their functionality via the well‐established angiogenesis assay.

### 2.5 Investigating and Testing Angiogenesis

**Figure 6:**
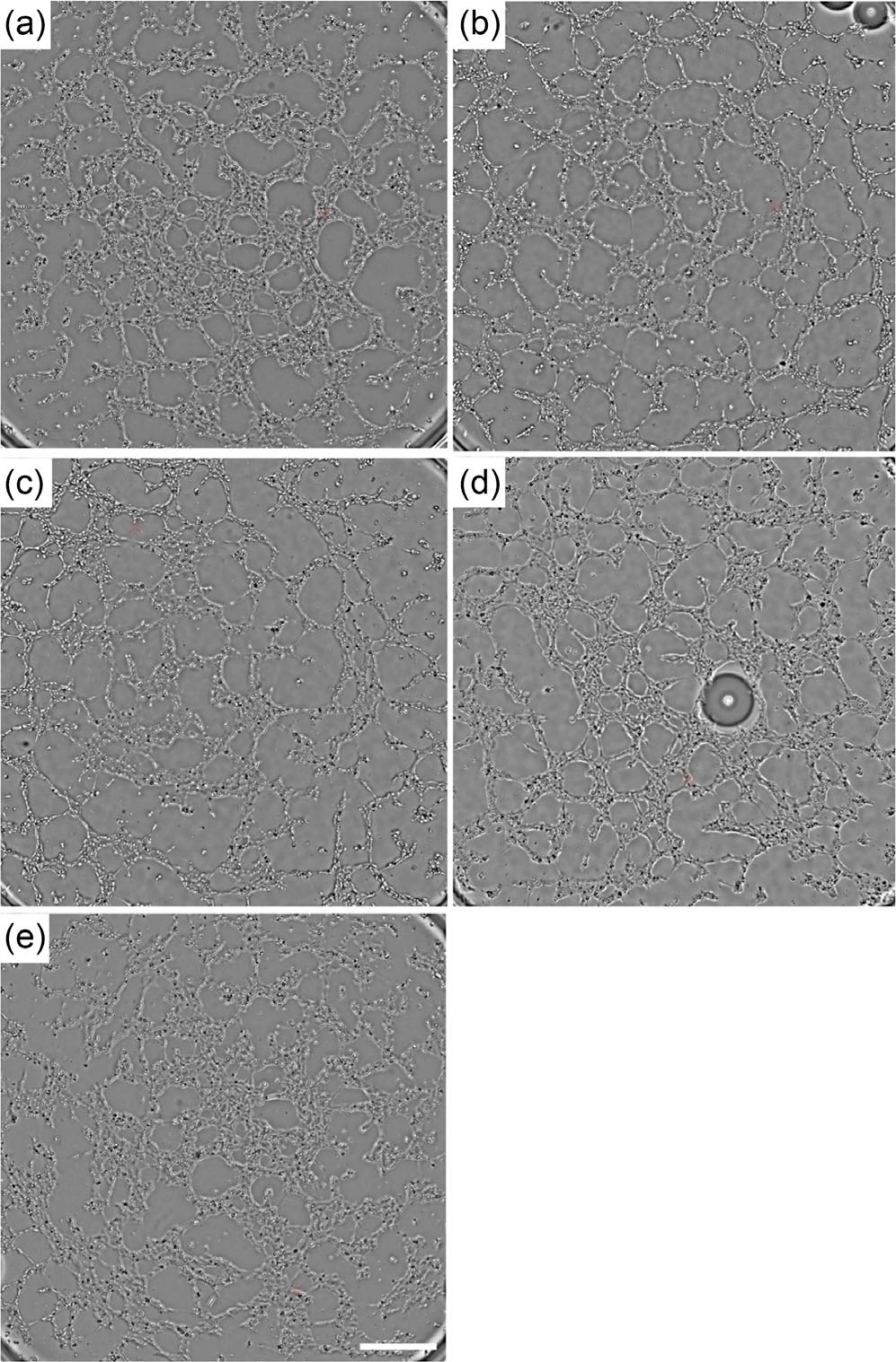
Characteristic microtubule networks generated from samples of as processed and collected, a) CC (suspension of HUVECs and PVA‐HUVEC prepared suspension), b)BE, c) CE, d) AABJ, and e) aerodynamically bio‐threaded over a time course of 24 hr postseeding. Scale bar in panel (e) is applicable to all panels and represents 500 μm.

In these studies, we not only wanted to assess cell viability over time both visually and through cytometry, but also wanted to interrogate the cell's functionality, to assess its ability to sprout and form microtubule networks (**Figure** **6**). For this, we followed the manufacture's protocol/application notes as published by ibidi (tube formation assay).^[^ ^19^ ^]^ Fresh HUVEC and PVA‐HUVEC samples were prepared and collected postprocessing and assessed for their angiogenic capacity. Figure 6 depicts representative microtubule networks formed from samples a) CC, b) BES, c) CE, d) AABJ, and e) AABT of those cells as processed and collected and subjected to the angiogenesis assay over a time course of 24 hr. We repeated the studies for samples from postprocessed samples at time points 24, 48, and 72 hr, and these samples were also seen to form microtubule networks, which were indistinguishable between samples at those respective time points.

The angiogenesis assay demonstrated (Figure 6), the cells capacity to undergo angiogenic sprouting and forming of microtubule networks was not affected by these bio‐jetting or threading processes.

### 2.6 Chicken Embryo Chorioallantoic Membrane (CAM) Assay Studies

**Figure 7:**
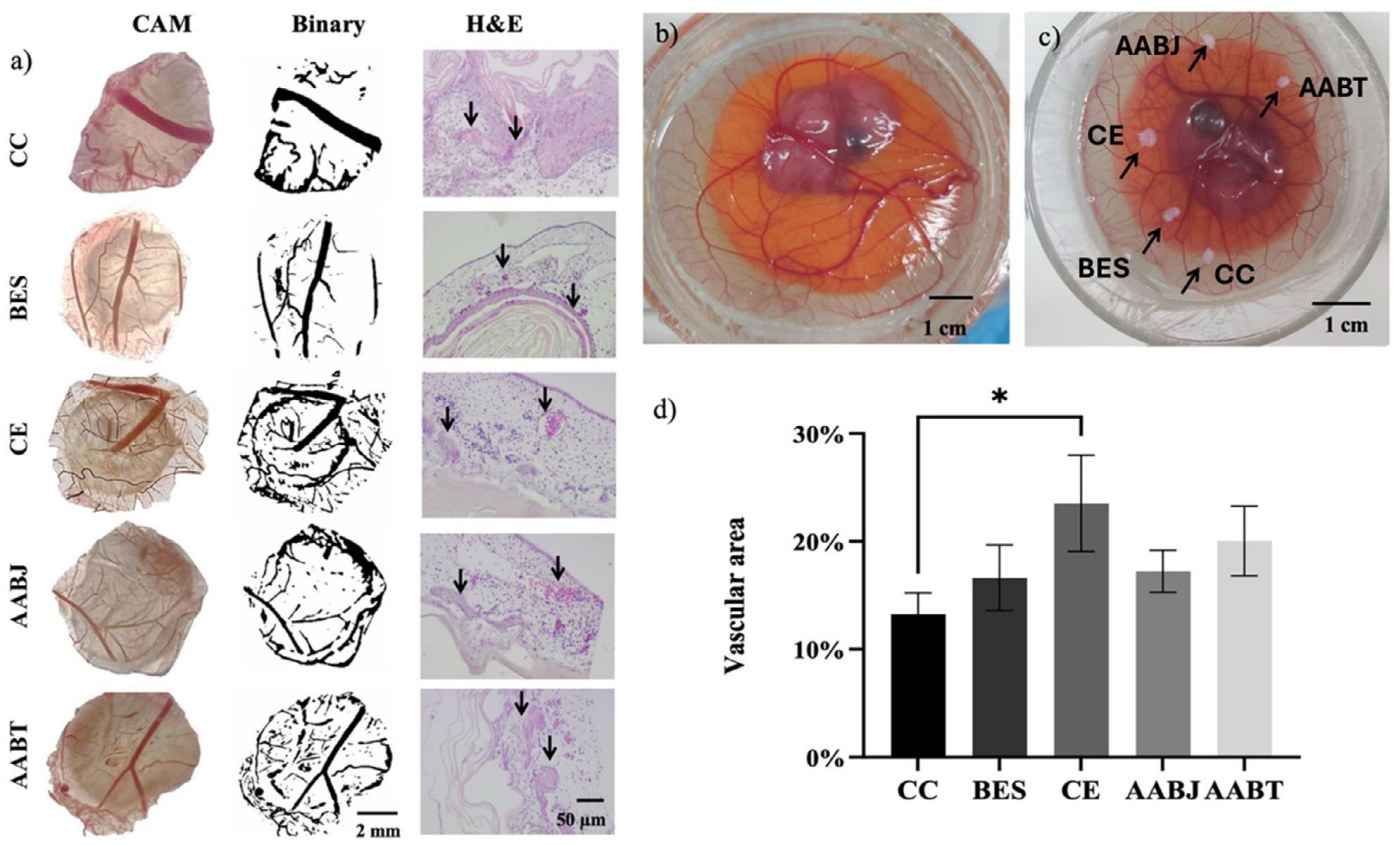
Showing results of vascularization on the scaffolds by CAM assay. a) Vascularized collagen scaffold by CC, BES, CE, AABJ, and AABT treatment with binary mode and H&E staining showing infiltration of blood vessels (black arrow) into scaffolds, b) developed CAM model by ED 10, c) implanted scaffolds on CAM at ED 13, black arrows identifies the samples (CC, BES, CE, AABJ, and AABT), and d) quantification of total vascular area% and * denoted statistical significance of *p* > 0.05.

Several HUVEC‐seeded cell scaffolds postjetting or ‐threading were placed onto the CAM assay,^[^ ^20^ ^]^ over several days. In this study, we assessed the angiogenic capacity of CC, BES, CE, AABJ, and AABT and total vascular area% in different experimental groups, showing considerable variations in angiogenic response (**Figure** **7**). At embryonic day (ED) 10 (Figure 7b), the different treatments generating five different HUVEC‐bearing scaffolds (CC, BES, CE, AABJ, and AABT) were transplanted onto the CAM assay. Figure 7c shows the transplanted scaffolds on ED 13.

In the CC group, the calculated total vascular area was 13.25 ± 1.99%, serving as a reference point for comparison. The observed level of vascularization in the untreated CAM tests was following the expected baseline, which serves as a benchmark for control. There was a 16.62 ± 3.03% increase in the total vascular area in the BES group. This suggests that applying an electric field while spraying promotes angiogenesis. An increase in the vascular area% suggests that BES has the potential to promote endothelial cell migration and proliferation, which is crucial for angiogenesis. The CE group also showed a significant increase in angiogenesis with a calculated vessel area of 23.51 ± 4.46% in the scaffold. This indicates CE's potential to promote angiogenesis. Therefore, an increase in significant vascular area% in the CE group showed that in CE, angiogenesis is upregulated by increased expression of growth factors or receptors involved in blood vessel formation. The implementation of AABJ treatment resulted in a vascular area of 17.24 ± 1.96% indicating a substantial angiogenesis stimulus. This suggests that the process of jetting efficiently promoted the formation of a vascular network. This can be mainly attributed to the precise delivery and distribution of cells and bioactive compounds. Furthermore, the AABT treatment similarly demonstrated an increase in angiogenesis with a total vascular area of 20.04 ± 3.23%. The presence of a vascular area in the AABT group suggests that the application of threading techniques allows accurate positioning of cells, promoting angiogenesis. This technique can be further developed by establishing nanoscale settings that promote cellular interactions and the deposition of materials outside of cells. Therefore, results indicated that each of the treatment groups (BES, CE, AABJ, and AABT) had higher angiogenesis than the CC, and the CE group exhibited the most significant effect (*P* < 0.05, Figure 7d). From panel a) in Figure 7, it is seen, that the vasculature had microintegrated effectively into and around the HUVEC‐bearing scaffold, forming blood vessels (indicated with black arrows in Figure 7a). This is a direct result of HUVECs being efficiently delivered deep into the open cellular structure of the generated collagen scaffolds by these microtechnologies. The results demonstrate that these microtechnologies can optimize angiogenesis, a crucial process for applications in tissue regeneration, repair, and other biomedical fields. The results of this study highlight the efficiency of different microtechnologies in affecting the angiogenesis response in the BES, CE, AABJ, and AABT groups. Thus, it is imperative to enhance these techniques in use in tissue engineering and regenerative medicine. Further investigation should be carried out to understand the mechanism of proangiogenic effects of these methods, as well as examine their enduring implications on the healthy and functional condition of tissues.

### 2.7 Immunostaining for CD31

**Figure 8:**
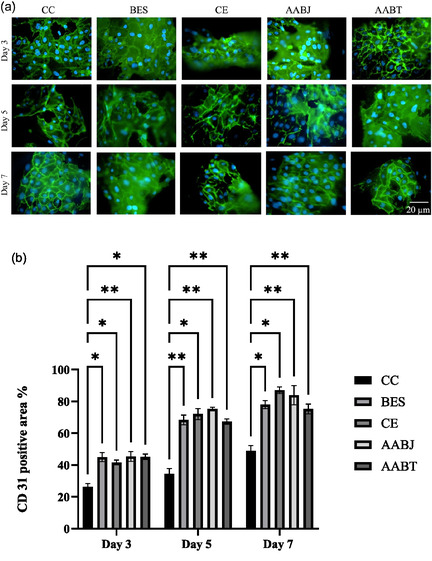
Panel a) representative figures showing CD 31 staining for CC, BES, CE, AABJ, and AABT treatment for day 3, 5, and 7. Scale bar is applicable to all panels. Panel b) illustrates the area% quantification of CD 31 staining for CC, BES, CE, AABJ, and AABT groups for days 3, 5, and 7 and * denotes the statistical significance of *P* < 0.05 and ** *P* < 0.01.

Angiogenesis was evaluated by CD31 immunofluorescence staining at 3, 5, and 7 days, respectively (**Figure** **8**). On day 3, the CC group exhibited a CD31‐positive zone that was relatively small and disorganized, suggesting baseline angiogenic activity in the seeded CC group. We observed an insufficient vascular network, indicating poor early‐stage angiogenetic response. In contrast, the group that received BES treatment exhibited a significant increase (*P* < 0.05) in CD 31 positive area as compared to the CC group. Additionally, we have observed an increased endothelial cell density and formation of early cellular networks, suggesting that BES promotes early angiogenesis. The cohort that received CE treatment also exhibited a significant increase (*P* < 0.05) in the surface area of CD31‐positive cells, characterized by the formation of broader and more interconnected networks results, suggesting that the CE group has a robust proangiogenic potential. Similarly, the group that had AABJ treatment displayed a significant increase (*P* < 0.01) in the CD31‐positive cells area with a stimulated proliferation of endothelial cells and the initiation of vascular network structure development. Furthermore, the AABT group, similar to the groups, showed a substantial increase (*P* < 0.05) in the CD31‐positive area compared to the CC group. Additionally, the AABT group displayed a well‐developed cellular network, indicating the effectiveness of AABT in promoting early angiogenesis.

On day 5, the CC group showed minimal alteration in the little modifications in the CD31‐positive region, indicating an absence of significant angiogenic activity over a period of time, and the microvasculature network was disorganized. On the other hand, the cohort that received BES treatment showed an increased in the area of CD31‐positive cells. This was accompanied by a proliferation of endothelial cells, indicating that BES has a sustainable proangiogenic impact. Similarly, the CE group showed a significant increase (*P* < 0.05) in CD31‐positive cell area as compared to day 3. Further, there was an increased complexity and expansion of vascular networks, further corroborating the potent proangiogenic effect of cell electrospinning. Similarly to both BES and CE, AABJ cells also demonstrated a significant increase (*P* < 0.01) in the CD31‐positive area, resulting in the development of organized and dense microvascular networks. This suggests a significant increase in the angiogenic response in comparison to day 3. We observed similar cellular behavior from AABT cells, which showed a significant increase (*P* < 0.01) in the area of CD31‐positive cells, indicating an increase in density and angiogenesis. This demonstrates that AABT remains effective in promoting the formation of new blood vessels over time.

On day 7, the CC group demonstrated negligible increases in the CD31‐positive region. The microvasculature was poorly organized, indicating minimal angiogenic activity. On the other hand, the BES group regularly demonstrated an expanding CD31‐positive area (*P* < 0.05), characterized by large and well‐developed vascular networks, indicating a continuous angiogenesis process. The group treated with CE had the highest density and complexity of CD31‐positive cell networks relative to the other cohorts (*P* < 0.05). This signifies a robust and continuous proangiogenic response during the study period. The AABJ group demonstrated additional improvement in the density and organization of the vascular network (*P* < 0.01). This signifies a robust angiogenic activity. Furthermore, the AABT consistently exhibited significant improvements (*P* < 0.01) in the area of CD31‐positive cells, distinguished by organized and dense vascular structures, hence demonstrating the persistent efficacy of promoting angiogenesis.

Overall, the CD31 immunostaining analysis findings on days 3, 5, and 7 elucidated how angiogenesis alters over time in response to different treatments. The result suggests that all treatment methods, including BES, CE, AABJ, and AABT, significantly enhance endothelial cell proliferation and vascular network formation compared to the control group. The CE group consistently demonstrated the highest density and complexity of CD31‐positive vascular networks at all time points across the treatments, suggesting that cell electrospinning fosters an optimal environment for endothelial cell growth and angiogenesis. The BES, AABJ, and AABT groups had significant proangiogenic properties, characterized by notable vascular network density and structure improvements throughout the study period. At this time, we hypothesize that the observed increase in angiogenesis of processed cells results from these technologies ability to distribute cells into the open structures of the porous collagen scaffold, thereby promoting and enhancing cell proliferation, attachment and angiogenesis utilizing the interconnections within the porous collagen scaffold. These findings underscore the potential of biomanufacturing techniques to promote angiogenesis, which is essential for tissue regeneration and several medical applications. We have commenced investigations into understanding the underlying mechanisms responsible for these proangiogenic effects, and currently, we are assessing the possible long‐term implications for tissue health and functionality. Therefore, examining the CD31‐positive areas at multiple time intervals will provide a deeper insight into the progression of angiogenesis across diverse treatment groups.

## 3 Conclusion

The investigative studies presented in this study, demonstrate that these microbiotechnologies have no negative effects on the jetted or threaded HUVECs. In fact, the processed cells are seen to have similar viabilities as those CC, which have not been exposed to any process other than cell culture. This was validated using a clinical readout, namely flow cytometry. In these studies, we also assessed the cell's functionality using well‐established assays such as the angiogenesis and the CAM assay. In the angiogenesis assay we found that the processed cells were indistinguishable with those controls as they were able to undergo all expected cellular characteristics, namely from sprouting to the formation of microtube networks. Through the CAM assay, we were able to see those seeded cells on collagen scaffolds, encouraging vascular growth in this alternative in vivo system. Finally, we used a ubiquitous marker (CD31) to observe vascularization of control and jetted cells onto scaffolds, which demonstrated the jetting and threading capabilities for distributing cells into the open porous structure, and was seen to enhance angiogenesis when compared with manually seeded cells. These studies further support these biomicrotechnologies for their application in reconstructing vascular grafts and tissues for regenerative biology and medicine.

## 4 Experimental Section

4.1

4.1.1

##### Bio‐jetting and Threading Equipment Setup

Bio‐electrospraying and cell electrospinning were carried out with the same single needle system. The needle had an inner bore and outer diameter of ≈2100 and ≈2500 μm, respectively. The inlet of the needle was connected to a 10 mL syringe via sterile silicone tubing and placed in a Harvard pump (Model type PHD 4400, HARVARD Apparatus Ltd., Edenbridge, UK). The pump was capable of supplying the cell suspension to the needle at a constant flow rate regime from 10^−12^–10^−8^ m^3^ s^−1^. The jetting needle was connected to a high‐voltage DC power supply capable of providing a + 30 kV (Model FP‐30, Glassman Europe Ltd., Tadley, UK). In the case of BES, a ring ground electrode was placed below the needle for CE. The ring ground electrode was replaced with a grounded metal mesh placed in a sterile glass petri dish. In these studies, the current was well below ≈50 nA. For both AABJ and AABT, a single needle device was explored, having an inner bore diameter of ≈2000 μm and an outer diameter of ≈3200 μm. This needle accommodated within the pressurized chamber was placed ≈300 μm above and in‐line with the exit orifice. The applied pressure to the chamber could be varied between 0.1–6 bar. However, in these experiments, we explored a pressure below ≈1 bar in the chamber. Samples were collected onto sterile stainless steel petri dishes and/or glass beakers respectively.

##### Preparation of Collagen Scaffold

A neutralized collagen solution was prepared by adding 18 mL (90%) rat tail collagen type I monomeric collagen solution of 2.16 mg mL^−1^ in 0.6% acetic acid with a total protein concentration of 0.2% w/v (First Link, Birmingham, UK). A pH indicator and 2 mL (10%) of 10X Minimal Essential Medium (Invitrogen, Paisley, UK) were added to this. To get an ideal pH, the mixture was neutralized with 5 and 1 M sodium hydroxide (Sigma‐Aldrich, Dorset, UK). In these studies, the postneutralized (step 2 in Figure 1), collagen solution was jetted via both electrosprays and AAJ into a sterile dish and incubated at 37 °C for ≈20 min. Subsequently, the collagen fibrillogenesis was frozen at −80 °C for ≈2 hr and lyophilized for ≈48 hr to form a porous sheet. Similar in some respects, freshly prepared collagen solutions were left to semigel (≈5 min postpreparation—step 3 in Figure 1) and then exposed to electrospinning and AAT. Threading was seen to form as a result of the gelation process. The collected threads on sterile dishes were subsequently incubated at 37 °C for ≈20 min and followed the same protocol as above, finally forming a porous sheet. All four samples on complete gelation were seen to form a white sheet of collagen. These sheets were then peeled off the petri dishes, and 6 mm scaffolds were cut out using a 6 mm biopsy punch. All 6 mm scaffolds were analyzed and subsequently characterized for their pore thickness and pore range using micro‐CT. These studies showed that the four processes explored for generating these scaffolds did not have significant differences between them. That being said, we found electrosprays to be the simplest approach to processing these scaffolds as AAJ, electrospinning and AAT of the collagen solution were found to leave residues at the needle exits, which over long periods were found to affect the spraying and spinning process. Figure 2a depicts characteristic high‐speed images captured during the electrospraying of the collagen solution, taking place in the unstable mode. Panels b–d in Figure 2 demonstrate a representative 6 mm scaffold generated via electrospraying and its porosity characteristics.

##### Scaffold Characterization

Scaffold samples were prepared for scanning using a high‐resolution micro‐CT scanner (Skyscan 1176, Skyscan, Belgium) with submicron resolution. The sections were scanned using an X‐ray voltage of 45 kV without a filter. The source current was 556 μA, with a rotation step of 0.5 degrees and an exposure period of 180 ms. The Skyscan NRecon programme (Bruker micro‐CT, Belgium) was utilized to reconstruct 3D models. The pore size and pore thickness were analyzed by processing the images using CTAn software (Bruker micro‐CT, Belgium) after performing segmentation and binarization. The 3D structure of the scaffolds was visualized using CTVox software (Bruker micro‐CT, Belgium) through volume rendering. This thorough imaging and analysis method enabled accurate characterization of the scaffold's microarchitecture, which is crucial for these studies.

##### Human Umbilical Vein Endothelial Cells (HUVEC) Culture and Suspension

HUVECs were commercially purchased by the previous working group (PromoCell,UK). Cryo‐vials of passage 3 were thawed, and cells were cultured in Endothelial Cell Growth Medium with supplements (PromoCell, UK) and 1% Pen‐Strep (Sigma, UK) at 37 °C with 5% CO2. All experiments were carried out at passage 5. The cell suspensions prepared for jetting and threading contained HUVECs suspended in Endothelial Cell Growth Medium with supplements (PromoCell, UK) and 1% Pen‐Strep (Sigma, UK) for BES and AABJ. For CE and AABT, the above cell suspension was mixed with sterile PVA at a concentration of 10%wt.

##### Cell Viability Assessment via Flow Cytometry

Cell viability of the five samples, namely CC (those samples only exposed to cell culturing), and those samples exposed to BES, CE, AABJ and AABT were collected and counted. For cytometry 1 × 10^6^ cells mL^−1^ were aliquoted and labeled respectively as per the manufacturer protocol (FITC Annexin V Apoptosis Detection Kit with PI, BioLegend, 33 Greenwood Place, London, NW5 1LB, UK), and interrogated via a BD LSR II (Wokingham, Berkshire RG41 5TS, UK) cytometer. Generated dot plots identified those cellular populations alive, early and later apoptosis to those dead cells.

##### Angiogenesis Assay

The angiogenesis studies carried out in these investigations explored cells from the five samples, namely CC, BES, CE, AABJ, and AABT with μ‐Slide 15 Well 3D plates (ibidi GmbH, 82 166 Gräfelfing, Germany). The angiogenesis and tube formation assay was setup as per the ibidi application note.^[^ ^19^ ^]^ Time‐lapse microscopy was carried out on a Nikon Ti2 microscope using a 4× NA0.13 PlanFluor objective and additional 1.5× tubelens. Cells were maintained at 37 °C and 5% CO_2_. Brightfield images were captured with a Photometrix Kinetix camera at 5 min intervals, Z‐stacks of 7 slices at 29 μm spacing were captured at each position. Stacks were processed into single in‐focus images using Gaussian‐Based Stack Focuser plugin in Fiji. Images shown were FFT bandpass filtered to increase contrast.

##### Chicken Embryo Chorioallantoic Membrane (CAM)

In this study, we assessed the angiogenesis ability of the scaffolds using a chorioallantoic membrane (CAM) experiment. We used a glass‐cling film CAM system that has been previously published.^[^ ^20^ ^]^ Pathogen‐free fertilized eggs were procured from Fosters Poultry (Gloucester, UK) for commercial use. These eggs were then placed in an incubator set at a temperature of 38 °C and a humidity level of 45–50%. On embryonic day (ED) 3, the eggs were opened and placed in an environment with 80–90% humidity at a temperature of 38 °C for further 6 days. On ED 10, five HUVEC‐bearing scaffolds (CC, BES, CE, AABJ, and AABT) measuring 6 mm in diameter and 1 mm in thickness were implanted into each CAM. At ED 13, scaffolds were harvested, and macroscopic photographs were captured. Postharvesting the scaffolds from CAM's the images were examined using ImageJ software (NIH). This analysis included converting the images to binary format and measuring % of the areas of vascular density. This approach facilitated an accurate evaluation of the scaffolds capacity to stimulate angiogenesis.

##### CD 31 Immunofluroscence Staining

Post‐treatment collagen scaffolds were fixed in 10% NBF after day 3, 5 and 7. Scaffolds were permeabilized in 1% TritonX‐100 solution for 15 min. A Rabbit polycloncoal 1:100 anti‐CD‐31 primary antibody (AbCam, UK) was used for immunostaining. After 2 h Alexa Fluor 488 ‐conjugated goat anti‐rabbit IgG (AbCam, UK)1:200 dilution in blocking buffer used as a secondary antibody for an hour, and DAPI was used as counter nucleus stain. All images were obtained via an Olympus BX43 fluorescence microscope at 20X.

##### Statistical Analysis

Statistical analysis was carried out using GraphPad Prism Version 10.4.1 (GraphPad Software Ltd, USA). All analyses were performed using a sample size of *n* = 3, representing three independent biological repeats for each condition, using two‐way ANOVA (multiple comparisons) with post hoc analysis. A *p*‐value < 0.05 was denoted by *, and a *p*‐value less than <0.001 was denoted as **.

## Author Contributions

**Prasad Sawadkar**: conceptualization, methodology, investigation, writing—review and editing. **Ayad Eddaoudi**: investigation, writing—original draft, writing—review and editing. **Dale Moulding**: methodology, investigation, writing—review and editing. **Suwan N. Jayasinghe**: conceptualization, methodology, writing—original draft, writing—review and editing.

## Conflict of Interest

The authors declare no conflict of interest.

## Data Availability

The data that support the findings of this study are available from the corresponding author upon reasonable request.
